# Basal Cell Carcinoma of the Head and Neck

**DOI:** 10.1155/2011/496910

**Published:** 2010-12-15

**Authors:** Masahiro Nakayama, Keiji Tabuchi, Yasuhiro Nakamura, Akira Hara

**Affiliations:** ^1^Department of Otolaryngology, Graduate School of Comprehensive Human Sciences, University of Tsukuba, 1-1-1 Tennodai, Tsukuba 305-8575, Japan; ^2^Department of Dermatology, Graduate School of Comprehensive Human Sciences, University of Tsukuba, 1-1-1 Tennodai, Tsukuba 305-8575, Japan

## Abstract

Basal cell carcinoma (BCC) is a malignant neoplasm derived from nonkeratinizing cells that originate from the basal layer of the epidermis and is the most frequent type of skin cancer in humans, with cumulative exposure to ultraviolet radiation as an important risk factor. BCC occurs most frequently at sun-exposed sites, with the head and neck being common areas. Tumors can be classified as nodular, superficial, morpheaform, infiltrating, metatypic, and fibroepithelioma of Pinkus. Several treatment options such as surgical excision and nonsurgical procedures are available. The choice of treatment should be determined based on the histological subtype of a lesion, cost, its size and location, patient age, medical condition of the patient, treatment availability, and the patient's wishes. The aim of any therapy selected for BCC treatment involving the head and neck is to ensure complete removal, the preservation of function, and a good cosmetic outcome.

## 1. Introduction

Basal cell carcinoma (BCC) is defined by the World Health Organization Committee on the histological typing of skin tumors as “a locally invasive, slowly spreading tumor which rarely metastasize, arising in the epidermis or hair follicles and in which, in particular, the peripheral cells usually simulate the basal cells of the epidermis” [1]. BCC constitutes approximately 75% of nonmelanoma skin cancers. It is usually observed in older patients, especially in those frequently and intensively exposed to ultraviolet radiation during their lives. The most typical site of BCC is uncovered skin directly exposed to the sun. Thus, BCC is often observed in head and neck areas, especially the eyelid and nose. It occurs chiefly in the elderly and is more common in males. Generally speaking, the tumor grows slowly and behaves in a nonaggressive fashion. BCC may be treated by surgery, cryotherapy, radiotherapy, and curettage and electrodessication [2]. Other less frequently used treatment modalities include the topical application of 5-fluorouracil (5-FU) ointment, laser treatment, and systemic chemotherapy [3]. To achieve a favorable outcome, it is important to recognize the histological subtypes, identify the anatomic locations that can increase the risk of spread, and understand the limitations of all available treatment modalities. If surgical defects are repaired, it is necessary to carefully plan the reconstruction after the tumor margins have been cleared. This paper discusses the histopathology, clinical presentation, and management of BCC of the head and neck. Local flaps for reconstruction of surgical defects are also described.

## 2. Etiology

The role of sunlight as a causative factor in cutaneous carcinoma is further reflected in its geographic distribution. There are more cases in southern compared to northern areas of the United States [4]. Individuals with more darkly pigmented skin have a lower rate of BCC, being rare in those of African descent [5]. This may be due to the protective effect of melanin pigmentation [4]. Embryonic fusion planes—the regions of mesenchymal migration and fusion of the five primordial facial processes during the 5th to 10th weeks of human development—have been implicated in the pathogenesis of basal cell carcinoma. Newman and Leffell [6] reported that basal cell carcinoma was more than four times more likely to occur on the embryonic fusion plane than on other regions of the midface.

There is a much greater incidence of BCC in males than females [4]. This may reflect a higher rate of sun exposure of males because of employment patterns. A population-based incidence study in Minnesota gave annual incidence figures for males and females of 175 and 124 per 100,000, respectively [7]. However, the incidence in women is increasing because of changing fashions in clothing and time spent outdoors due to recreation patterns or specific occupations. It has been suggested that the incidence of persons affected by BCC is likely to substantially underestimate the true incidence of this cancer [8]. This is due to the fact that BCCs are not routinely registered because of their high frequency and low mortality. In addition, occurrence of multiple primary tumors within individuals synchronously or at different times is common in BCC patients. Australian surveys demonstrated that the incidence of people treated for new primary BCCs was 1.5% in 10 years [8] and that over 700 persons per 100,000 person years were affected by multiple BCCs [9].

BCC is more frequent in the elderly, and the incidence of BCC increases with age. More than 90% of BCCs are detected in patients aged 60 and older [10–13].

## 3. Clinical Features

BCC growth is characteristically slow, often evolving for months to years. The actively growing tissue is at the periphery of the lesion, with cellular apoptosis and resultant ulceration in the central region. To treat these lesions, it is important to eradicate the farthest marginal areas because these tend to have the most aggressively behaving cells. Growth may continue for months or even years, gradually invading and destroying bone as well as soft tissues. There is a predilection for invasion along tissue planes, the periosteum, and nerves. A common theory states that the embryonic fusion planes, such as the nasolabial fold, are more susceptible to tumor growth.

## 4. Diagnosis

Dermoscopy is a noninvasive technique that is known to increase the diagnostic accuracy of benign versus malignant pigmented skin lesions [14–19].

**Figure 1:**
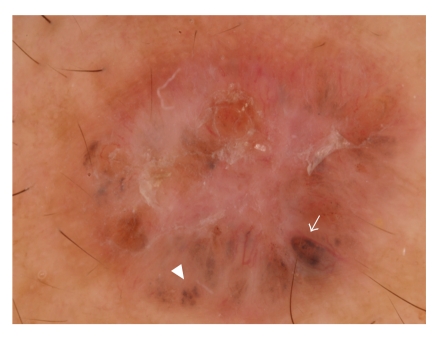
Representative photograph of dermoscopy. Morphologic features of BCC, such as telangiectasia (arrow), blue-gray globules (arrowhead), and ulceration, are seen.

Menzies et al. [20] recently proposed a simple dermoscopic model for the diagnosis of pigmented BCC, based on the absence of a pigment network and the presence of at least one of six positive morphologic features. Positive dermoscopic features include ulceration, multiple blue-gray globules, leaflike areas, and telangiectasia. Furthermore, large blue-gray ovoid nests have been defined as pigmented ovoid or elongated areas, larger than globules, and not intimately connected to the pigmented tumor body. Spoke wheel areas are an additional parameter appearing as well-circumscribed radial projections, usually tan, but also blue or gray, meeting at an often darker (dark brown, black, or blue) central axis (Figure 1) [20].

 Dermoscopy is frequently able to differentiate between BCC and other pigmented skin lesions, such as malignant melanoma and seborrheic keratosis.

## 5. Histopathology

BCC is characterized by large nuclei that are oval and composed mostly of cellular matrix, with little cytoplasm. There is a higher nucleus-to-cytoplasm ratio in malignant compared with normal cells. Tumor masses are surrounded by a peripheral cell layer in which the nuclei form a palisade or picket fence-type arrangement. Tumors can be classified as nodular, superficial, morpheaform, infiltrating, metatypic, and fibroepithelioma of Pinkus [21]. Histopathological types of BCC have been associated with different results and prognoses. 

**Figure 2:**
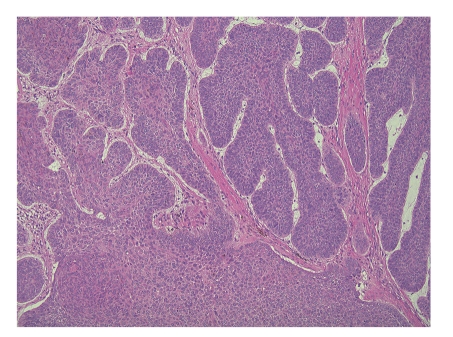
Microphotograph of nodular basal cell carcinoma (hematoxylin and eosin (HE) staining). Note tumor cells with large and hyperchromatic ovoid nuclei and little cytoplasm showing peripheral palisading.

Nodular BCC is the most frequent form of BCC, accounting for 75% of all cases, being superficial or ulcerated and often visualized on actinic damaged skin [22]. This lesion often shows slow growth. Further, around 90% of nodular BCC lesions are found on the head and neck (Figure 2).

**Figure 3:**
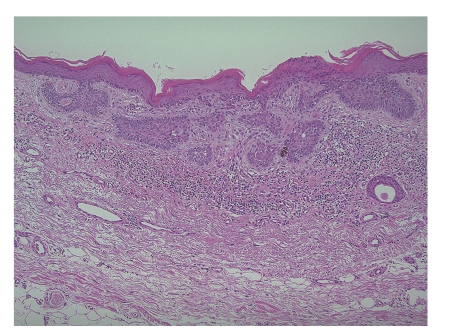
Microphotograph of superficial basal cell carcinoma (HE staining). The histologic characteristics of superficial BCC include buds and irregular proliferations of tumor cells attached to atrophic epidermis.

Superficial BCC appears as a plaque or as an erythematous squamous plaque, often found on the trunk and extremities, although 40% still occurs on the head and neck [22] (Figure 3).

**Figure 4:**
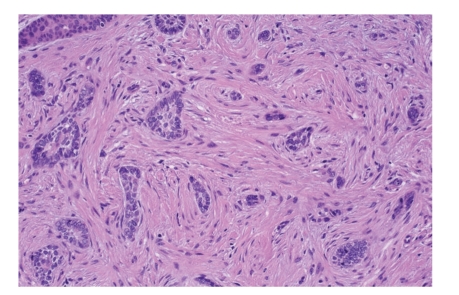
Microphotographs of morpheaform BCC (HE staining). Morpheaform BCC is characterized by its deep invasion of the dermis. The overlying skin surface may be atrophic, ulcerated, or relatively normal in appearance.

Morpheaform BCC accounts for approximately 6% of all BCC, but 95% of these will be located on the head and neck [22, 23]. It tends to be more aggressive, sometimes infiltrating deeper in muscles or fat tissue (Figure 4). It clinically resembles a scar or a small patch. There are no sites of predilection, and these lesions rarely bleed or get ulcerated.

Infiltrating BCC has been linked to morpheaform or nodular BCC. Metatypic BCC shows clinical signs of BCC as well as squamous cell carcinoma (SCC). This subtype tends to be more aggressive than the other subtypes, and it could grow and extend as SCC does, with a marked presence of metastasis. Fibroepithelioma of Pinkus often appears in the lumbar and resembles a fibroepithelial polypus or seborrheic keratosis [24, 25].

## 6. Treatment

Common treatments for BCC of the head and neck include methods such as Mohs micrographic surgery, surgical excision, liquid nitrogen cryosurgery, and curettage and electrodessication. Other less frequently employed treatment modalities include the topical application of 5-fluorouracil (5-FU) ointment, laser treatment, radiotherapy, and systemic chemotherapy [3]. The choice of treatment should be determined based on the histological type of lesion, cost, its size and location, patient age, medical condition of the patient, treatment availability, and the patient's wishes. The aims of any therapy for the treatment of a BCC are to ensure complete removal, the preservation of function, and a good cosmetic outcome. 

### 6.1. Mohs Micrographic Surgery

Mohs micrographic surgery is well established as the standard of care in many cases of BCC and squamous cell carcinomas. In 1941, Frederick Mohs described a surgical technique he had developed for the staged removal of skin cancer using *in situ* fixation of cutaneous tissue using a paste containing zinc chloride [26]. In 1953, he used frozen section without chemical fixation to excise a recurrent tumor on the eyelid [27]. Tromovitch and Stegman [28] reported the fresh tissue technique. They noted less pain, discomfort, and anxiety in patients treated with this technique compared with those treated with chemical fixation [27, 28]. A detailed map of the tumor site was made to record the positive margins and to direct the next excision. Despite advances in techniques, the basic principles have remained the same in that histologically examined tissue directing further resection until all margins are clear of tumor.

 There are several advantages to performing Mohs micrographic surgery to the treatment of BCC [29]. First, Mohs micrographic surgery is the most effective method of eradicating BCC, with a five-year cure rate of 99 percent [30–32]. Another benefit is that Mohs micrographic surgery spares tissue. Preserving uninvolved tissue is of paramount importance, especially around the eyes, nose, ears, mouth, and genitalia. Finally, compared with other surgical techniques involving postoperative repair, the cost of Mohs micrographic surgery is similar to that of simple excision in the office with permanent section postoperative margin control. Mohs micrographic surgery is less expensive than excisions with intraoperative margin control with frozen sections performed in a private office or in an outpatient surgical facility [33].

### 6.2. Surgical Excision

Treatment requires total excision of the lesion. Surgical excision facilitates pathologic assessment of the tissue [34, 35]. The surgical specimen should be oriented for the pathologist so that the margins can be examined, allowing the surgeon to verify residual tumor presence or complete excision. It is very important to achieve adequate surgical margins [36]. The margins will depend on the size of the lesions, anatomic location, clinical features, ulceration, and apparent depth of penetration. It has been common practice to employ a 5 mm margin for excision around BCC [4]. Some authors suggested that surgical margins of less than 5 mm might be adequate for noninvasive small BCC of the head and neck. Wolf demonstrated that margins of 4 mm were adequate in 95% of nonmorpheaform BCC less than 2 cm in diameter when treated by standard excision [37]. In addition, Lalloo and Sood [2] reported that a clinical excision margin of 2 mm was adequate for the treatment of simple, well-demarcated BCCs arising in the head and neck except for recurrent or morpheaform tumors. While these margins are adequate for a small BCC whose histologic subtype is such that the tumor does not warrant marked lateral or deep excision, it is not an acceptable margin for large tumors or lesions exhibiting a morpheaform histology [4]. 

 Silverman et al. [38] analyzed 588 primary and 135 recurrent BCCs treated by surgical excision. Primary-treated tumors had a cumulative 5-year recurrence rate of 4.8%, whereas recurrent tumors recurred at a rate of 11.6%, showing a statistically significant difference. The recurrence rate is higher for head and neck tumors, with that of the ear being the highest [38]. There was no significant difference with regard to the size of the primary lesion. However, Dubin and Kopf [39] reported that BCC recurrence rates increased with an expanding lesion size. Lesions smaller than 2 mm did not recur, lesions 6 to 10 mm showed a recurrence rate of 8.8%, and lesions larger than 30 mm recurred 23.1% of the time. As described above, BCC often originates in the skin of the nose, eyelid, or ear. Surgeons should be familiar with the structures of these lesions and also with the reconstruction methods following the surgical resection of deep penetrating lesions.

### 6.3. Surgical Excision of Nose Lesions

The nose is a common location of BCC tumors. Numerous reconstruction methods have been devised and utilized according to the characteristics of the defect. Primary closure is the most straightforward method if the defect is small. Although skin grafting is a simple option, it is not a suitable reconstruction method for most defects of the nose, because it is difficult to obtain a good texture and color match. A local flap is a more favorable reconstruction method for the lower portion of the nose, where the skin is thick and dense with sebaceous glands [40]. However, if a defect of the nasal tip or the ala is superficial and too large to cover with a local flap, a full-thickness skin graft can be used, especially when the skin is relatively thin and sebaceous glands are sparse [41]. 

Some local flaps often used for reconstruction of the nose are listed as follows.

**(1) Nasolabial Flap**

**Figure 5:**
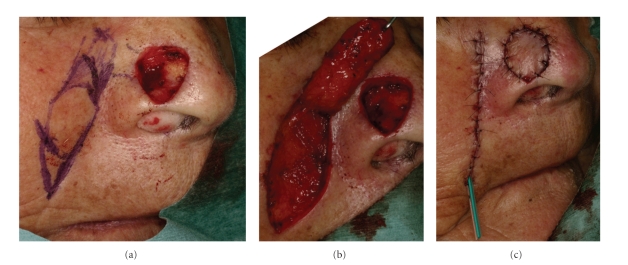
Nasolabial flap. (a) A flap is designed on the nasolabial fold. (This is permitted by Japanese Dermatological Association). (b) The flap is elevated in the subcutaneous plane. The flap is rotated and transferred to the defect. (This is permitted by Japanese Dermatological Association). (c) The skin defect of nasolabial fold was closed directly. (This is permitted by Japanese Dermatological Association).

The superiorly based nasolabial flap is useful for defects of the nasal sidewall, ala, and tip, while the inferiorly based nasolabial flap is useful for defects of the upper and lower lip, nasal floor, and columella [41]. An interpolated design is cosmetically desirable. The blood supply to this flap is excellent due to perforating branches of the facial artery. The color and texture are excellent matches, while the donor site scar is acceptable in the nasolabial sulcus. Using a template defect, a flap is designed on the nasolabial fold (Figure 5(a)). It is best to make the flap exactly match the defect size. The medial incision for the flap follows the nasolabial sulcus, and the lateral incision is placed no higher than the level of the inferior defect margin. The flap is elevated in the subcutaneous plane, and the plane goes deeper as it proceeds superiorly (Figure 5(b)). The flap is rotated counterclockwise on the right side and transferred to the defect [41] (Figure 5(c)).

**(2) Subcutaneous V-Y Flap**

Sliding, subcutaneous V-Y flaps for the reconstruction of nasal defects have been gaining in popularity, especially in nasal dorsum reconstruction. The flaps have also been used in ala nasi reconstructions, for defects generally limited to less than 1.5 cm in diameter and not involving the rim [42]. The advantages of having similar tissue in the same operative field, with an excellent blood supply, make the V-Y flap a common choice for nasal reconstruction. The area of lesion excision and the flap is marked preoperatively. Once all margins are known to be clear after tumor excision, the V-Y flap is dissected out and moved inferiorly on a subcutaneous pedicle to repair the defect. However, this flap has limitations, particularly in instances involving the inferior margins of the nose near the anterior nares. Some notching along the alar rim may occur and, in younger individuals, would probably be severe. For repair of the nostril rim, this flap may not be effective. The higher the defect is located on the nostril away from the rim, the easier the reconstruction is and the more favorable the result is [42].

**(3) Bilobed Flap**

The bilobed nasal flap is a useful and time-honored technique for reconstructing defects of the nose, especially defects of the lower third of the nose [41, 43]. The bilobed flap is appropriate for partial-thickness losses of less than 1.5 cm of the lateral aspect of the nose, ala, and tip area. This flap is essentially a rotation flap divided into two transposition flaps, with an excellent blood supply from angular and supraorbital arteries. It recruits skin from the middorsum and sidewall.

**Figure 6:**
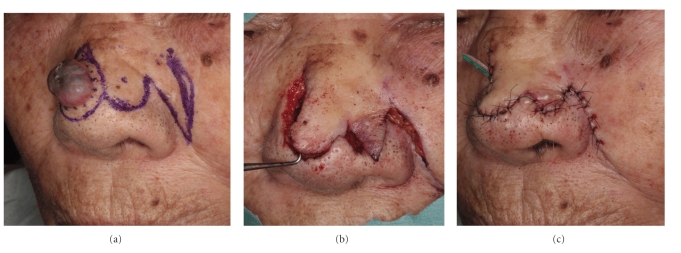
Bilobed flap. (a) Two flaps and surgical margin (5 mm) were designed. (b) The primary flap closes the tumor defect, and the secondary flap is used to close the donor site. (c) The donor site of the second lobe is closed primarily.

The two flaps have a common base and typically form an arc of no more than 90–110° to avoid tension development on wound closure (Figure 6(a)). The angle between the defect and first lobe is equal to that between the first and second lobe [41]. The size of the first lobe equals that of the defect, and the second lobe is 2/3 the size of the first lobe. The primary flap closes the tumor defect, and the secondary flap is used to close the donor site (Figure 6(b)). The donor site of the second lobe is primarily closed [43] (Figure 6(c)).

**(4) Midline Forehead Skin Flap (Seagull Flap)**

The midline forehead skin flap can serve as a cover for any nasal reconstruction from severe tip and ala loss to a total nasal defect. Using this flap, esthetic and functional reconstruction can be achieved by creating a nose that blends well with the face. The seagull-shaped flap is based on one of the supratrochlear vessel bundles. Its vertical axis is placed over the midline of the forehead, and the wings are designed to lie in natural transverse creases. Local flaps are turned over and carried down to the septal support for lining. The forehead flap is elevated and transposed 180° to cover the new nose. The body of the seagull lies along the bridge, the wings curl at the ala and turn into the nostril sills, and the seagull head and neck creates the tip and columella. The forehead donor site is primarily closed [44].

### 6.4. Surgical Excision of Eyelid Lesions

The eyelid is also a common location of BCC tumors. BCC accounts for 90 to 95% of malignant eyelid tumors [45–47]. Regarding periocular BCC, lower eyelid lesions are the most common, accounting for up to two-thirds of cases, followed by the upper eyelid, medial canthus, and lateral canthus [45–47]. Although small partial-thickness eyelid defects may be closed by simple suture, reconstruction of the lower eyelid after surgical excision is quite challenging. Salomon et al. [48] reported that local flaps or full-thickness skin grafts should be recommended in cases of small- and medium-sized lower eyelid skin defects. They also reported that the bilobed flap seemed to be the most appropriate among numerous possible regional flaps for small- and medium-sized lesions. The nasolabial flap can also be used for partial or total lower eyelid reconstruction. The flap is based superiorly, so that it can easily be rotated to the lower eyelid position. Larger defects of the medial canthus and adjacent eyelids may be covered with midline forehead transposition flaps [48]. Full-thickness palpebral defects ranging from one quarter to one-half of the lower eyelid may be repaired easily with the use of a cheek rotation flap [48]. 

 A deep penetrating lesion of the eyelid may invade the orbit and/or paranasal sinuses. In such cases, orbital exenteration and/or resection of the paranasal sinuses may be required [47–49]. In a study of invasive BCC, Leibovitch et al. [47] suggested that medial canthus BCC posed a higher risk of orbital invasion.

### 6.5. Surgical Excision of Ear Lesions

A wedge excision of the auricle with a margin is often used for auricular BCC. The wound edges can be closed without tension in small lesions. A variety of local flaps for external ear and conchal reconstruction have been described, and full-thickness skin grafts (FTSG) have been used as well [50]. Dessy et al. [50] reported that their first choice for the skin graft donor site is usually the contralateral postauricular area. 

 After BCC removal from the external auditory canal, closure of the skin defect of the external auditory canal may not be needed if the underlying bone of the external ear canal is intact. To cover the exposed bone of the external auditory canal (the skin defect), a skin graft from the postauricular area may be a possibility. A conchal skin flap may also be selected. Based either inferiorly or superiorly, the entire conchal skin and subcutaneous tissue can be elevated and transposed across the meatus of the external auditory canal. The flap donor site is usually closed with a skin graft [51].

### 6.6. Curettage and Electrodesiccation

Curettage and electrodesiccation (CE) comprise one of the most frequently used treatment modalities for BCC. The gross tumor is removed with a curet, and the base is desiccated with a cautery. The advantages of this technique are that it is quick and straightforward to learn. The disadvantages are that, without biopsy and specimen orientation, histological control is poor or absent, and hypertrophic scars and hypopigmentation may occur. 

It is generally accepted that when effectively treated by CE experts, cure rates of more than 95% can be expected for appropriately selected BCC [52]. The types of BCC that should not be treated by CE include large, infiltrating, morpheaform, and recurrent tumors [52]. Silverman et al. [53] demonstrated that larger lesions, the diameter, and high-risk anatomic sites were independent factors affecting the recurrence rate (RR). In their study, BCC treated with CE at low-risk sites (neck, extremities) had a cumulative 5-year RR of 3.3% for lesions of any diameter. At medium-risk sites (scalp, forehead, auricular, and malar), BCCs with diameters of less than 10 mm led to a 5-year RR of 5.3%, whereas those of 10 mm or larger had a higher RR of 22.7%. At high risk sites (nose, nasal labial groove, canthi, and ear), BCCs of less than 6 mm in diameter led to a 5-year RR of 4.5%, whereas those of 6 mm or greater were associated with a 5-year RR of 17.6%. Telfer et al. [52] reported that all primary BCC patients with tumor of facial sites exhibited a 5-year cumulative RR of 1.2%, with 3 recurrences (nose, eyelid, and preauricular region) in 256 patients.

### 6.7. Cryotherapy

Cryotherapy is destructive modality that has been used in the treatment of BCC [54, 55]. Two freeze-thaw cycles with a tissue temperature of −50°C are required to destroy BCC. Cryotherapy lacks the benefit of being able to histologically confirm confirmation of tumor removal. Kuflik and Grage [56] reported 99% cure rates in 628 patients followed for 5 years. August [57] suggested that cryotherapy should be avoided for the scalp and nasolabial fold sites because of the high rate of recurrence of the tumor. Ceilley and del Rosso [58] also mentioned that aggressive cryotherapy may induce tumor recurrence because of concealment of the tumor by a fibrous scar. Nakai et al. [59] reported a case of a nodular BCC with a skip lesion on the nose, near the nasolabial fold, after repeated cryotherapy. As for cryotherapy, continuous and careful observation of the clinical course is required.

### 6.8. Radiotherapy for Head and Neck BCC Lesions

Radiotherapy can yield a high cure rate for BCC, and adjunctive radiotherapy can improve local regional control in cancer with adverse features such as the presence of perineural spread, extensive skeletal muscle infiltration, bone or cartilage invasion, and positive nodal/extranodal spread [60]. Lauritzen et al. [61] reported that the cure rate with radiotherapy was 92.7% at 5 years in a series of 500 BCC patients. Seegenschmiedt et al. [62] reported that complete remission was achieved in 99% of patients by 3 months after treatment, in 127 BCC lesions of the head and neck region. Swanson et al. [63] reported that radiation therapy for BCC of the medial canthus resulted in a 100% control rate for positive margins and a 92% control rate for gross disease. 

 Regarding disadvantage of radiotherapy, radiotherapy may cause common cutaneous side effects such as acute and chronic radiation dermatitis [64, 65]. Good initial cosmetic results can deteriorate with time, such that skin may show poikiloderma. It is desirable to avoid radiation therapy in young patients because of the late effects of irradiation [66].

### 6.9. Photodynamic Therapy (PDT)

PDT exerts its local effects via light-dependent cytotoxicity. The treated area is exposed to monochromatic light after local or systemic administration of a chemical photosensitizer, such as methyl aminolevulinate. The wavelength matches the absorbance peaks of the photosensitizer used. The photosensitizer absorbs light energy and then interacts with reactive oxidative species or directly with cellular substrates, resulting in cell death via apoptosis or necrosis [67]. Good treatment results of PDT were reported in superficial and nodular BCCs with response rates of 85%–92% in superficial BCC and 73%–91% in nodular BCC [68–74]. Although longer followup studies are required, reported data indicate the potential of PDT as a noninvasive treatment alternative for superficial and nodular BCCs [74].

### 6.10. Hedgehog Signaling Pathway Inhibitors

BCC is associated with mutations in components of the hedgehog signaling pathway [75]. Mutations in hedgehog pathway genes, primarily genes encoding patched homologue 1 (PTCH1) and smoothened homologue (SMO), occur in BCC. Von Hoff et al. [76] assessed the safety and pharmacokinetics of GDC-0449 (vismodegib), a small-molecule inhibitor of SMO, and responses of metastatic or locally advanced basal cell carcinoma to the drug. They reported that 18 of 33 BCC patients had a response to GDC-0449 and that only one grade 4 adverse event occurred during continuous daily administration of GDC-0449 for up to 19 months. GDC-0449 is currently undergoing phase II clinical trials for the treatment of advanced BCC. Because of its low toxicity and specificity for the hedgehog pathway, this drug has potential advantages compared with conventional chemotherapy and may also be used in combination treatments [77].

## 7. BCC Syndrome

Nevoid basal carcinoma syndrome, also referred to as Gorlin-Goltz syndrome or basal cell carcinoma syndrome, is a rare autosomal dominant disease showing a genetic predisposition characterized by multiple BCC [78–81]. 

 Patients with BCC syndrome show multiple abnormalities, none of which are unique to this syndrome [80, 81]. The three abnormalities traditionally considered to be the most characteristic of the syndrome are BCC, pits on the palm and sole, and cysts of the jaw. Palmoplantar pits are small defects in the stratum corneum and may be pink or, if dirt has accumulated, dark in color. Jaw cysts are often the first detectable abnormalities, and they may be asymptomatic and, therefore, diagnosed only radiologically. However, they also may erode enough bone to cause pain, swelling, and loss of teeth. A minority of BCCs demonstrate aggressive behavior and involve the craniofacial bones in nevoid BCC syndrome. Tabuchi et al. [49] reported a nonfamilial case of nevoid BCC syndrome with a BCC of the eyelid invading the ethmoid sinus. 

 Because the individual abnormalities are not unique to BCC syndrome patients, it is possible to clinically diagnose BCC syndrome only when multiple, typical defects are present. The severity of abnormalities may differ markedly among members of a single family, and diagnosis certainly may be difficult in individuals. Generally, the diagnosis is suggested in a patient with BCC arising at an unexpectedly early age and in unexpectedly large numbers [49]. The gene for BCC syndrome has been mapped to chromosome 9q22.3-q31 [82, 83]. Two researchers have independently demonstrated that BCC syndrome is caused by mutations of the PATCHED1 (PTCH1) gene [82, 83].

## 8. Conclusions

BCC is more common than all other cancers, and the most frequently seen malignancy by most doctors regardless of their specialty. We have to recognize BCC and its different histologic subtypes, as well as areas in which these might occur. Several treatment options such as surgical and nonsurgical are available. Mohs micrographic surgery is the standard treatment for cases of BCC on the head and neck. Defects after surgical excision may be repaired by adequate local flaps. Radiation therapy is also used in the treatment of primary BCC or in cases where postsurgical margins are positive for cancer. Adequate treatment promises superior local disease control for BCC. Thus, doctors in all specialties need to become more aware of BCC, and accurate and early diagnoses need to be made by them.
